# Effect of Honey/PVA Hydrogel Loaded by Erythromycin on Full-Thickness Skin Wound Healing in Rats; Stereological Study

**DOI:** 10.31661/gmj.v0i0.1362

**Published:** 2019-04-16

**Authors:** Shabnam Movassaghi, Zahra Nadia Sharifi, Mojtaba Koosha, Mohammad Amin Abdollahifar, Shahrzad Fathollahipour, Javad Tavakoli, Shabnam Abdi

**Affiliations:** ^1^Department of Anatomical Sciences & Cognitive Neuroscience, Faculty of Medicine, Tehran Medical Sciences, Islamic Azad University, Tehran, Iran; ^2^Department of Cellulose and Paper Technology, Faculty of New Technologies Engineering, Shahid Beheshti University, Zirab Campus, Savadkooh, Mazandaran, Iran; ^3^Department of Biology and Anatomical Sciences, School of Medicine, Shahid Beheshti University of Medical Sciences, Tehran, Iran; ^4^Department of Chemical and Biomolecular Engineering, University of Akron, Ohio 44325, USA; ^5^Mechanical Engineering Biomechanics and Implants Research Group, The Medical Device Research Institute (MDRI), School of Computer Science Engineering and Mathematics, Sir Eric Neal Building, Flinders University, Adelaide, Australia

**Keywords:** Erythromycin, Honey, Polyvinyl Alcohol, Wound Healing

## Abstract

**Background:**

Skin wounds are a significant public health risk, and treatment of wound remains a challenging clinical problem for medical teams and researchers.

**Materials and Methods:**

In the present study, we aimed to investigate the healing effects of honey/polyvinyl alcohol (PVA) hydrogel loaded with erythromycin as wound dressing on skin wounds in rats, based on histological studies. In this study, 60 male Wistar rats, with a 1.5 ×1.5 cm2 diameter full-thickness wounds on the backs were divided into four groups: honey/PVA with the erythromycin hydrogel group, honey group, PVA group, and the control group, with no treatment. Skin biopsies were prepared at days 4, 7, and 14 for microscopic analyses. The stereological analysis, including the mean area of the wound, length of vessels, numerical density of fibroblast, macrophage, basal cell and volume of the epidermis, dermis, and fibrous tissue were performed.

**Results:**

Wounds area in the honey/PVA hydrogel with the erythromycin group were significantly (P<0.05) smaller than in the other group. The numerical density of fibroblast, macrophage, basal cell and volume of the epidermis in the honey/PVA hydrogel with the erythromycin group were significantly higher than other groups.

**Conclusion:**

According to our results, honey/PVA hydrogel with erythromycin may promote early wound healing and has a positive influence on fibroblast proliferation and re-epithelialization, and its administration is recommended after further validation of clinical data.

## Introduction

The human skin is the largest organs in the body that easily injured by many harmful external factors, such as an incision, trauma, burns, lacerations, or abrasion [1]. In the clinic, skin wounds represent an extremely common and major unresolved problem in modern medicine that affects many of people in the world and induce high socio-economic costs for therapy in many cases [2]. Wound healing is a dynamic and complex process that requires the interactions among different cell types, growth factors, and extracellular matrix and consists of three phases, including inflammation, proliferation, and remodeling [3, 4]. Many attempts have been made in several studies, focusing on the principles and mechanisms of the wound healing, to accelerate the repair process and develop new therapeutic approaches that promote cell adhesion and the proliferation of the healing process. However, conventionally wound healing management approach, using natural or synthetic bands, cotton, and linen gauze, still not successful due to ischemia and bacterial infection [5, 6]. In recent decades, close attention has been made to a biomaterial scaffold that promotes wound repair [7-9]. Polyvinyl alcohol (PVA) is one of the most clinically effective materials among novel types of wound healing products. PVA-based hydrogels are hydrophilic, non-toxic, non-carcinogenic, biodegradable, biocompatible, water-soluble, and cost-effective polymer. It exhibits permeability to small molecules, and have good physical, gas permeation and transparency that make it desired wound dressing materials [10]. The use of hydrogels based on natural saccharides and disaccharides, such as honey and sucrose can increase mechanical strength, biocompatibility, and water uptake of the wound dressing [11, 12]. Honey as natural substances has been traditionally used to stimulate wound regeneration and has pharmacological characteristics, including antioxidant, anti-microbial, and anti-inflammatory effects [13-16]. The combination of PVA and honey (H/PVA) interestingly has been used in the wound; however, development of a wound dressing of H/PVA hybrid hydrogel is difficult, due to structural instability and low mechanical strength of hydrogel. In other hand, high temperature during crosslinking of hydrogel reduces honey antibacterial properties. Also, many studies stated that PVA hydrogels have the potential to be used as controlled-release drug delivery systems carrying drugs [6, 17]. Erythromycin as an antibiotic agent with a short half-life is commonly used for the treatment of bacterial infections of skin [18, 19]. In this regard, synthesis of PVA loaded with erythromycin can be used for delivery of erythromycin through a wound dressing to prevent wound infection and minimize the undesirable side effects of the erythromycin, like abdominal cramps, vomiting, and diarrhea. To the best of our knowledge, the combination of H/PVA and erythromycin has not been developed so far and here, for the first time we are examining the wound effect of this new mixture. The current study aimed to find the impact of H/PVA hydrogels loaded with erythromycin on the full-thickness skin wound healing process in laboratory rats, and characterized, using stereological analyses to solve the above-mentioned problems for the first time.

## Materials and Methods

### 1. Drugs and Chemicals

PVA (M_w_ = 145,000; >99% hydrolyzed) and ethanol 99% were supplied from Sigma-Aldrich (USA). The pure dried honey was purchased from herbal medical store (the purity of the honey was 90%). Erytrimacyn was obtained from AFA Chemie pharmaceutical company (Tehran, Iran). Double distilled water (DDW) was used as solvent for preparation of solutions.

### 2. H/PVA Hybrid Film Preparation

PVA solution with a concentration of 10% (w/v) was prepared by adding PVA powder to DDW with constant stirring at 100±2°C for 2 hours at 1400 rpm in a sealed container. Honey was dissolved in DDW to prepare a solution of 15% (w/v). The honey and PVA solutions were mixed at 400 rpm for about 1 h in room temperature. Honey solution was added to PVA solution to reach a concentration of 1.5% (w/w) of honey in PVA and stirred at 1000 rpm for one hour at 80°C to get a hybrid homogeneous mixture. Equal weights of each mixture were molded into poly (styrene) Petri dishes and the solvent was allowed to evaporate at laboratory temperature for 48 to 72 h resulting in dry hydrogel films. The samples were treated at 80±2°C in an oven for 12 hours to achieve cross-linked hydrogels. During the fabrication process, a predetermined amount of the drug in the PVA hydrogels was loaded according to erythromycin pharmacodynamic and pharmacokinetic characteristics. To make solutions containing 1% erythromycin, appropriate amounts of a 10% solution of erythromycin were dissolved in ethanol 70% to the H/PVA solution and then casted in Petri dishes. The drug-containing solutions were heated at 50±2°C for 12 hours to achieve physically cross-linked hydrogels. Before further use, the hydrogel films were kept in desiccators. The physicochemical, mechanical and in vitro biocompatibility evaluation of the hydrogel films as wound dressing materials were performed in our previous work (Iranian Journal of Pharmaceutical Research, under review).

### 3. Animals

Sixty adult male Wistar albino rats weighing 250–300 g were purchased from Pasteur Institute of Iran, located in city of Tehran). The animals were kept in a standard animal housing facility room temperature (22–24°C), at animal research laboratory and free access to water and food. The Ethics Lab Animal Care Committee of the Islamic Azad University Tehran Medical Sciences Branch according institutional guidelines for the ethical care of animals approved the animal experiment (approval no. IR.IAU.TMU. REC.1395.393).

### 4. Experimental Procedure

**Figure 1:**
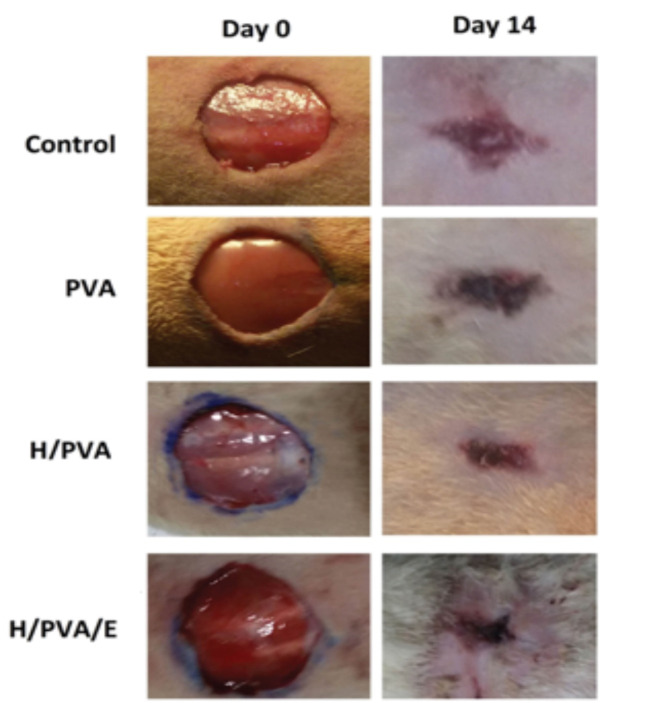
Representative images of wounds on the backs of the rats (dimension 1.5 × 1.5 cm2). In control, PVA, H/PVA, and H/PVA/E groups after 0 and 14 days of study.

After hairs shaving of the flanking region of the animals, an area of uniform wound (1.5×1.5 cm^2^, Figure-1) scratched using an upstanding bistoury and a full-thickness skin wound was created on dorsal skin of the rats under 50 mg/kg of ketamine (Alfasan^TM^, Woerden, Holland; 0.04 mL/100g body weight) and 5 mg/kg Xylazine (Alfasan^TM^, Woerden, Holland; 0.02 mL/10g body weight). Solution anesthesia to create excision wound model [20, 21]. After washing of the wounds with saline solution, the wounds were treated with the hydrogel/honey with and without erythromycin. The animals randomly were grouped into four groups (n=15 per group). One group was treated with PVA as base group. The second group was treated with H/PVA, and the third group was treated with H/PVA with erythromycin (H/PVA/E), and finally the control group received no treatment, and their wounds were kept without dressing. Then, the whole abdomen of animal was protected with gauze stretching bandages to keep dressings and each rat was allowed to recover in a separate cage. The hydrogels and bandages were changed every 3 days. For histological evaluation of wound healing process macroscopic analysis of the wounds was carried out at days 4, 7, and 14 and the photograph taken used for the area. After periods of 4, 7 and 14 days, the animals were sacrificed with a high dose of ether and skin with the wounds was removed from each experimental group and fixed in 4% paraformaldehyde solution for histological processing and stereological evaluations.

### 5. Estimation of the Wound Area

The images of skin wound in each group of study every four days were captured (n= 5 in each subgroup) with a digital camera (Samsung NX2000). To calculate its surface area accurately of each wound a standard ruler was set at the level of the wound at the same magnification and the photos were prepared and imported into Image J software (1.34u, National Institutes of Health, USA); using this software, the mean pixel /cm^2^ was determined, then, surface area of each wound represented as cm^2^.

### 6. Soteriological Study

Fifteen µm slides were provided by microtone from the paraffin blocks and stained with Hematoxylin and Eosin (H&E) and evaluated by stereological methods for volume of epidermis, dermis, and fibrous tissue, number of fibroblast, macrophage and basal cells and length of vessels.

#### 6.1. Estimation of the Volume of Epidermis, Dermis, and Fibrous Tissue

The tissue samples were fixed in 10% formalin for one week. Following tissue processing, serial coronal sections of 10µm thickness were prepared and stained with H&E. The total volume of epidermis, dermis, and fibrous tissue were measured by the Cavalieri method [22]. Thus, 8–10 sections were selected, using a systematic uniform random sampling. The image of each section was evaluated, using the stereological software and a video-microscopy system, made up of a microscope (Nikon, E-200, Japan), linked to a video camera. The volume of the testis was estimated by the following formula [22].

$$V_{total}=\sum P\times t\times \frac{a}{p}$$

In this formula Σ_P_ is the total points, hitting the testis sections, $\frac{a}{p}$ is the area associated with each point, and t is the distance between the sampled sections.

#### 6.2. Estimation Of The Number Of Fibroblast, Macrophage And Basal Cells

The total number of the fibroblast, macrophage, and basal cells was determined using the optical disector method. The positions of the microscopic fields were selected by an equal interval of moving the stage and systematic uniform random sampling. Microcator was used for measurement of Z-axis movement of the microscope stage. An unbiased counting frame with inclusion and exclusion borders was superimposed on the images of the sections, viewed on the monitor. A nucleus was counted if it was placed completely or partially within the counting frame and did not reach the exclusion line. Numerical density (N_v_) was calculated with the following formula:

$$N_{\nu }=\frac{\sum Q}{\sum P\times h\times \frac{a}{f}}\times \frac{t}{BA}$$

Where “Σ_Q_” is the number of the nuclei, “Σ_P_” is the total number of the unbiased counting frame in all fields, “*h*” is the height of the dissector, “ $\frac{a}{f}$” is the frame area, “t” is the real section thickness measured in every field using the microcator, and “BA” is the block advance of the microtome, which was set at 10μm. The total number of the neuron and glial cells was estimated by multiplying the N_v_ by the total V [10].

$$N_{total}=N_{\nu }\times V$$

#### 6.3. Estimation of the Length of Vessels

Each sample slide was analyzed with a video-microscopy system (Nikon E-200, Japan) linked to a video camera. The evaluation of the length of vessels under the microscope is an application of length estimation in two-dimensional planes. The following formulae use for estimation the length of the vessel [22].

$$L_{\nu }=\frac{2\times \sum Q}{\sum P\times \frac{a}{f}}$$

Where Σ_Q_ is the total number of the vessel profiles counted in each group; ($\frac{a}{f}$) is the area of the counting frame, and Σp is the total number of frames counted per animal.

### 7. Statistical Analysis

The study data were statistically analyzed using SPSS version 18.0 (Chicago, IL, USA) and represented as mean ± SD, and statistical significance was analyzed using one-way analysis of variance (ANOVA) and Tukey’s post hoc test between treated and control groups. P≤ 0.05 was considered as statistically significant.

## Results

### Area of the Wounds

**Figure 2:**
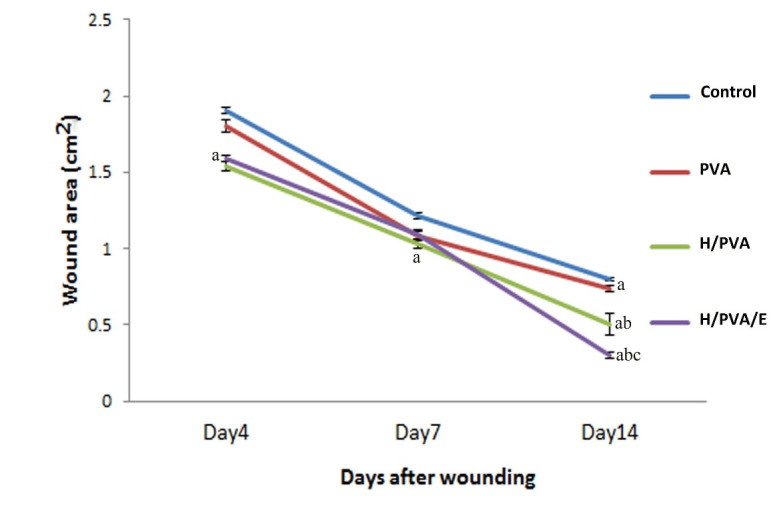
The area of the wound in the control, PVA, H/PVA, and H/PVA/E treated groups. Values are given as mean±SD. ^a^P<0.001 *vs.* control group; ^b^P<0.001 *vs.* PVA group

The average area of the wounds on days 4, 7, and 14 in each group was calculated and represented as mean ± SD (Figure-2). The primary wound surface area was 1.84± 0.01 cm^2^. A significant difference was found among the H/PVA/E with PVA and control groups at days 4, 7, and 14. There was significantly different between H/PVA/E and H/PVA at day 14 (P<0.05).

### The Total Volume of the of Epidermis, Dermis and Fibrous Tissue

**Figure 3:**
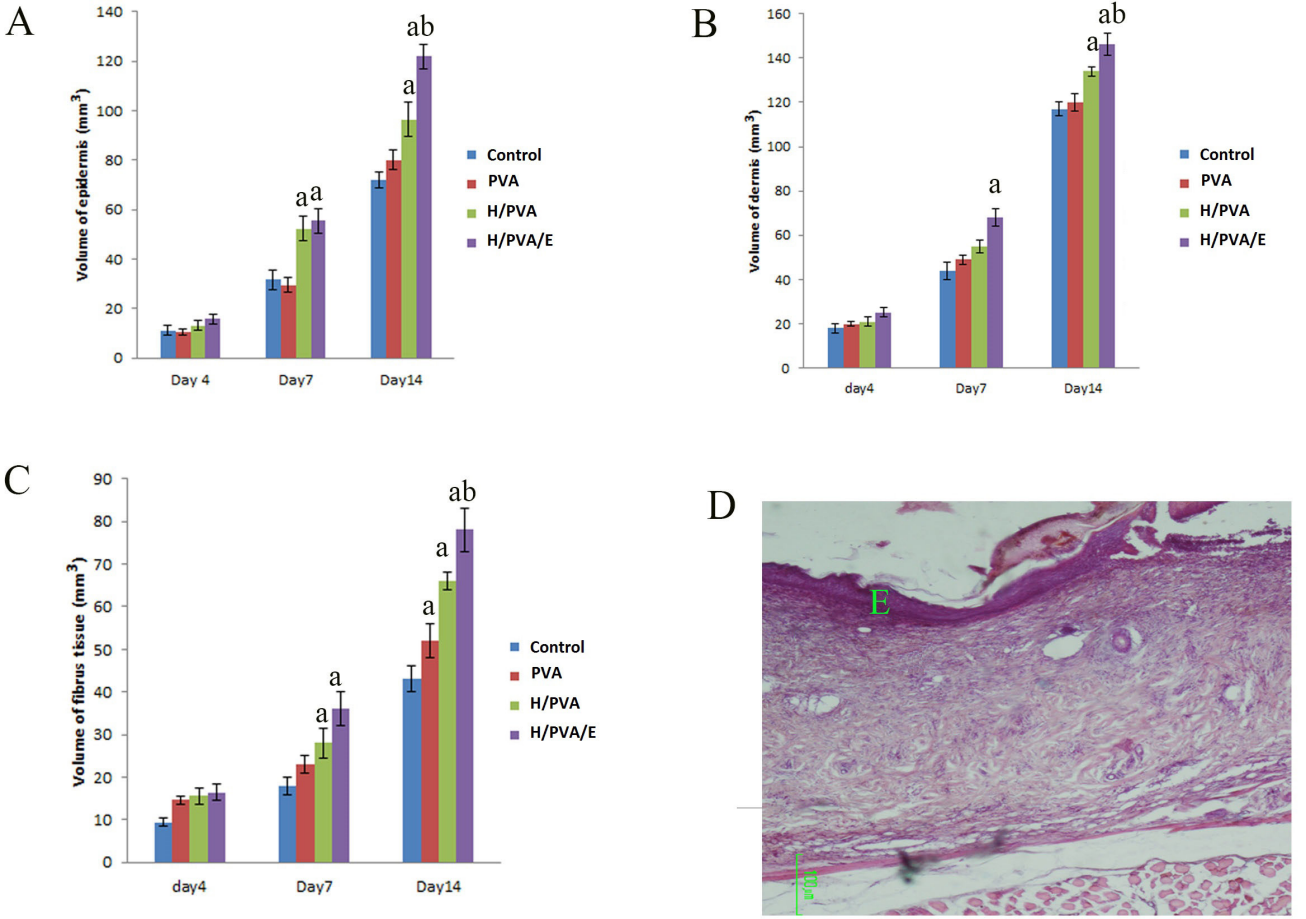
The total volume of the epidermis **(A)**, dermis** (B)** and fibrous **(C)** tissue in PVA, H/PVA, H/PVA/E, and control group were compared. Hematoxylin and eosin-stained histopathological view of the wound** (D)** at day 7 (40 × magnification). ^a^P<0.005 *vs.* control group; ^b^P<0.001 *vs.* PVA group

**Figure 4:**
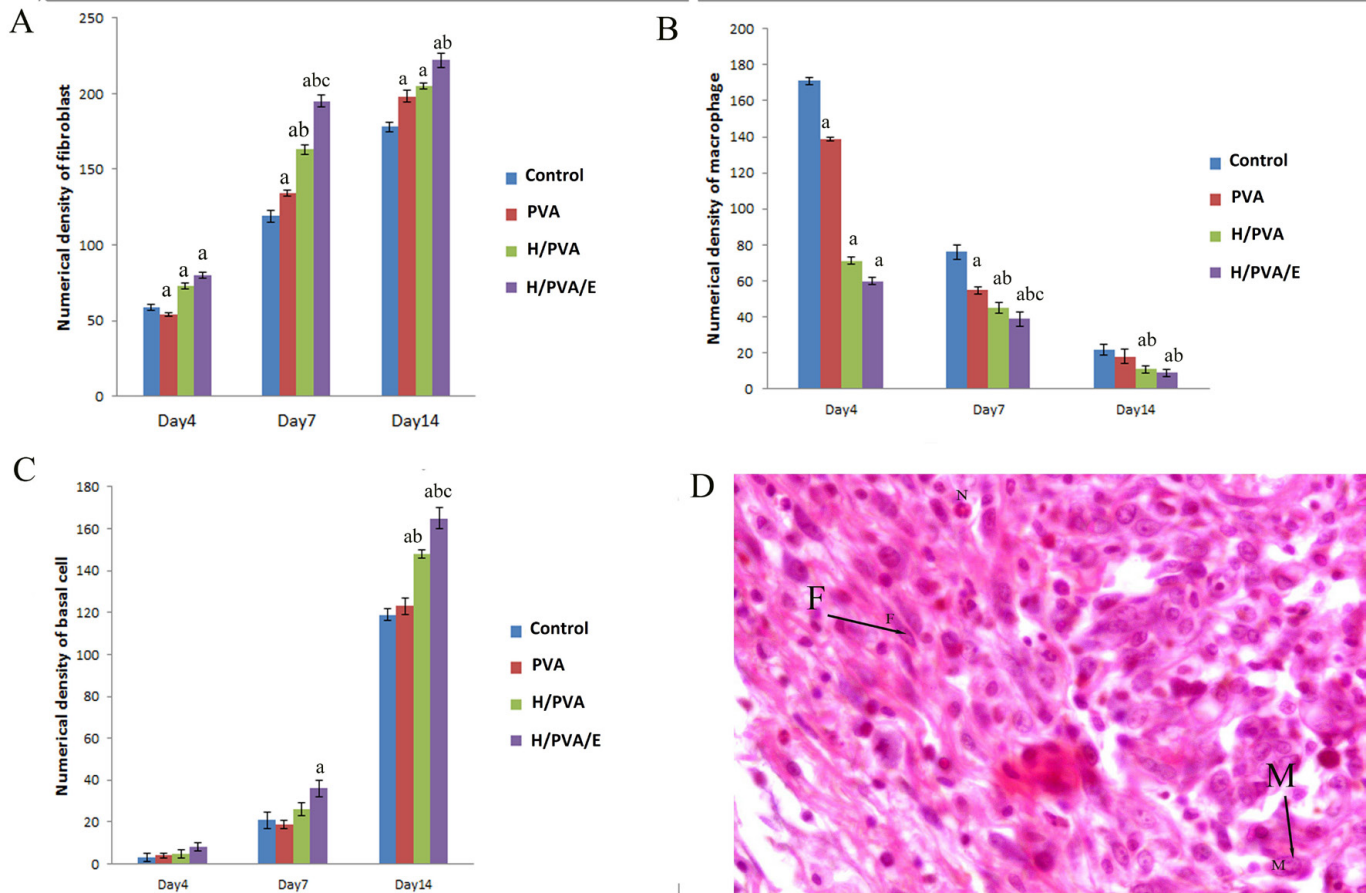
The numerical density of the fibroblasts **(A)**, macrophage **(B)** and basal cell** (C)** of the wounded rats treated with PVA, H/PVA, H/PVA/E, and untreated wounded group. Histology image **(D)** of the healed wound with fibroblast (**F** and arrow) and macrophage (**M** and arrow; H&E stainig, 40 × magnification). ^a^P<0.005 *vs.* control group; ^b^P<0.002 vs. PVA group; ^c^P<0.001 H/PVA group.

**Figure 5:**
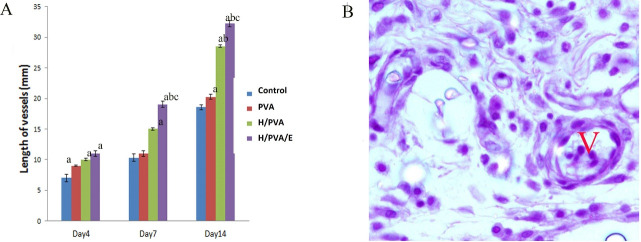
The length of the vessels in each group of study. **B:**Histology image of the vessel in the healed wound (arrows). ^a^ P< 0.005 vs. control group; ^b^ P<0.002 vs. PVA group; ^c^ P< 0.001 H/PVA group.

The total volume of the epidermis was significantly different in the H/PVA/E group in comparison to the PVA and control groups (P<0.05 and P<0.01, respectively). The volume of the fibrous tissue in the H/PVA/E group was significantly higher in comparison to the other group at day 14 (P< 0.001). There was a considerable difference between the total volume of the of the dermis and fibrous tissue in the group treated with H/PVA/E with other group at day14, and their data were summarized in Figure-3. Fibroblast, macrophage, and basal cell population were summarized in Figure-4. The N_v_ of the fibroblasts in the dermis of the H/PVA/E and H/PVA was significantly higher than that of the PVA and the control groups at days 7 and 14 (P=0.004). The number of fibroblasts in the H/PVA/E group and H/PVA group had a significant difference (P<0.05). The number of macrophages in the H/PVA/E group and H/PVA group had an insignificant difference at days 7 and 14. The numbers of macrophages in the H/PVA/E and H/PVA group significantly lower than PVA and control groups. The numbers of basal cell in the H/PVA/E group was markedly higher in comparison to that of the other group at day 14 (P < 0.001, Figure-4). However, significant differences in the length of vessels between the H/PVA/E and other groups were observed at day 21 (Figure-5). Also, the length density of the vessels was higher in H/PVA/E group, in comparison to the H/PVA (P=0.02).

## Discussion

This study demonstrated that H/PVA/E enhances proliferation of fibroblast, collagen synthesis, and revascularization and it showed anti-inflammatory impacts in the healing process of skin wounds. Further, the obtained results suggest that complete re-epithelialization with thick dermis and fibrous tissueformation occurs in H/PVA/E group, whereas the absence of compact collagen deposition in PVA alone and the control group was observed and in the control group, inflammatory cells appeared at day 7. Wound healing is divided into three stages of inflammation, proliferation, and remodeling. A few hours after injury, the inflammatory phase begins. The neutrophils and macrophages play important roles in this phase for the induction of fibroblast proliferation and formation of new blood vessels [23]. Our result showed that the number of macrophages in the H/PVA/E and H/PVA group were significantly lower than other groups. This could be related to the antibiotic effect of erythromycin and honey. Erythromycin as an antibiotic is useful for the treatment of some bacterial diseases, and in higher concentration inhibits the development of bacteria, and by releasing, a constant amount of erythromycin to the wound area can avoid any infection. Our results showed that the combinations of honey and erythromycin improved the associated strong antibacterial effect, which may be due to a potent synergistic effect. The mechanism of antimicrobial action of honey is correlated with high osmolality, acidic PH, the presence of hydrogen peroxide inhibitors, phenolic substances and flavonoids. Hydrogen peroxide with its insulin-like properties in honey is released slowly into the wound bed and induced cell proliferation, leading to angiogenesis in the wound bed [24]. On the other hand, honey with antioxidant activity prevents the generation of free radicals, and accelerate the inflammation phase [13, 15]. Our result indicated that H/PVA when combined with erythromycin, not only promotes antibacterial and anti-inflammatory activity in wound dressings but also demonstrates the high levels of vascularization and promotes fibroblast proliferation. In the proliferation phase, fibroblasts cells are activated in the wound bed. The significant increase of fibroblast cells in the H/PVA/E group at the days 7 and 14 of the study showed a positive effect of honey and erythromycin in PVA wound dressing on the proliferative phase. This resulted in faster wound healing and indicated the earlier beginning of a proliferation phase in H/PVA/E groups, while the PVA alone and the control group was still in the inflammation phases. The smaller wound area and complete repair of the wound after 14 days of dressing, confirmed the normal progress of healing after dressing with the H/PVA/E. The main reason for the wound size reduction in H/PVA/E group is the increase of the collagen fibers due to the high number of fibroblast. Consistent with our results, Majtan et al. reported that the honey could increase the proliferation of fibroblasts, in comparison to the control group [25]. Takzaree et al. show that using honey twice a day on open full-thickness wounds will accelerate the healing process. Another study shows that the number of fibroblasts, macrophages, neutrophils, vessels number, and collagen fibers had a significant difference in the experimental groups and control group [26]. Kujumgiev et al. showed that the use of propolis with antibiotics, improved its antimicrobial effect and it seemed that they had a synergistic effect [27]. In another study, Yasasvini et al. reported that loading simvastatin-chitosan microparticles into PVA hydrogels as a drug delivery system enhances wound healing rate [28]. On the other hand, one of the important factors in the wound healing process is angiogenesis. The growth of blood vessels in H/PVA/E groups was accompanied by an increasing trend in oxygenation and endothelial proliferation, in comparison with the control group and finally improves wound healing. As mentioned before, the properties of PVA make it a suitable material to exudate adsorption of the wound, prevent the bacterial penetration, and regulate the wound moisture and the proliferation [29]. It was observed that the H/PVA loaded with erythromycin led to the sustained release of honey and erythromycin, and the adhesion to the wound bed. Hence, we are reporting for the first time a novel scaffold that has been created by PVA to improve poor stability and solubility of honey to ameliorate the application on the wound and the release of erythromycin into the wound.

## Conclusion

Our study demonstrates that the H/PVA with erythromycin accelerated wound healing process by increased epithelization, shortened inflammatory phase, and revascularization and allowing the progress of a normal healing path and inducing the faster formation of new tissue with good histological features in comparison to an undressed wound. More scientific investigations and clinical studies are more strongly warranted to determine the role of H/PVA scaffolds in regulating another pro-inflammatory cytokines mediators and growth factors in wounds to determine its adverse effects and extend its use in clinics.

## Acknowledgment

This project was supported and funded by Islamic Azad University, Tehran Medical Branch (grant No. 601013163331).

## Conflict of Interest

There are no conflicts of interest.
